# 2186. Treatment-emergent resistance to ceftazidime-avibactam (CZA) is more common than ceftolozane-tazobactam (CT) among patients infected with multidrug-resistant (MDR) *Pseudomonas aeruginosa*

**DOI:** 10.1093/ofid/ofad500.1808

**Published:** 2023-11-27

**Authors:** Sunish Shah, Ghady Haidar, Kevin M Squires, Ellen G Kline, Jason M Pogue, Erin K McCreary, Madison Stellfox, Daria Van Tyne, Ryan K Shields

**Affiliations:** Antibiotic Management Program, UPMC Presbyterian Hospital, Pittsburgh, PA, Pittsburgh, Pennsylvania; University of Pittsburgh School of Medicine, Pittsburg, PA; University of Pittsburgh, Pittsburgh, Pennsylvania; University of Pittsburgh, Pittsburgh, Pennsylvania; University of Michigan, College of Pharmacy, Ann Arbor, Michigan; UPMC, Pittsburgh, PA; University of Pittsburgh, Pittsburgh, Pennsylvania; University of Pittsburgh School of Medicine, Pittsburg, PA; University of Pittsburgh, Pittsburgh, Pennsylvania

## Abstract

**Background:**

CZA and CT are front-line agents for treatment (tx) of MDR *Pseudomonas aeruginosa*; however, comparative data are lacking. The purpose of this study was to compare rates of tx-emergent resistance among patients (pts) with bacteremia or pneumonia at a single center.

**Methods:**

Adult pts treated for >48 hours with CZA or CT were included. Pts with cystic fibrosis or colonization were excluded. Isolates were tested by broth microdilution (BMD) in triplicate and underwent whole-genome sequencing.

**Results:**

113 pts were included. Demographics, severity of illness, and durations of tx were similar for pts tx’d with CZA or CT (**Table 1**). CT-treated pts were less likely to receive prolonged infusions and monotherapy, but more likely to have empyema/endovascular infections. Baseline median (range) MICs were 4 (2–8mg/L) and 2 (0.25–8mg/L) for CZA and CT, respectively. Within 90-days from tx onset, rates of resistance defined as either ≥4-fold MIC increase or MIC >8mg/L were higher among CZA-treated pts (**Table 1**). Corresponding median MICs of resistant isolates were 16 (16–128mg/L) and 64 (32–512mg/L), respectively. Across 107 baseline isolates, 59 different sequence types (ST) were represented. The most common *P. aeruginosa* cephalosporinase (PDC) variants were PDC-3, PDC-5, and PDC-8, present in 10%, 21%, and 10% of isolates, respectively. Resistance evolved across varying STs. A subgroup of contemporary pts was well-balanced and supported overall findings (**Table 2**). From this subgroup, rates of tx-emergent resistance among pts receiving CZA or CT monotherapy were 50% (6/12) and 6% (1/17), respectively (*P*=0.01). Rates of CT-resistance were lower among pts who received prolonged (5%, 1/19) versus standard (20%, 5/25) infusions. Paired baseline and resistant isolates showing ≥4-fold MIC increase were compared (**Table 3**). CZA resistance was associated with sequence changes in *ampD* and/or efflux genes in 5 pts; 2 additional pairs showed mutations in *ftsI* (PBP3). CT resistance was associated with new *ampC* mutations in 83% of pairs.

**Figure:**
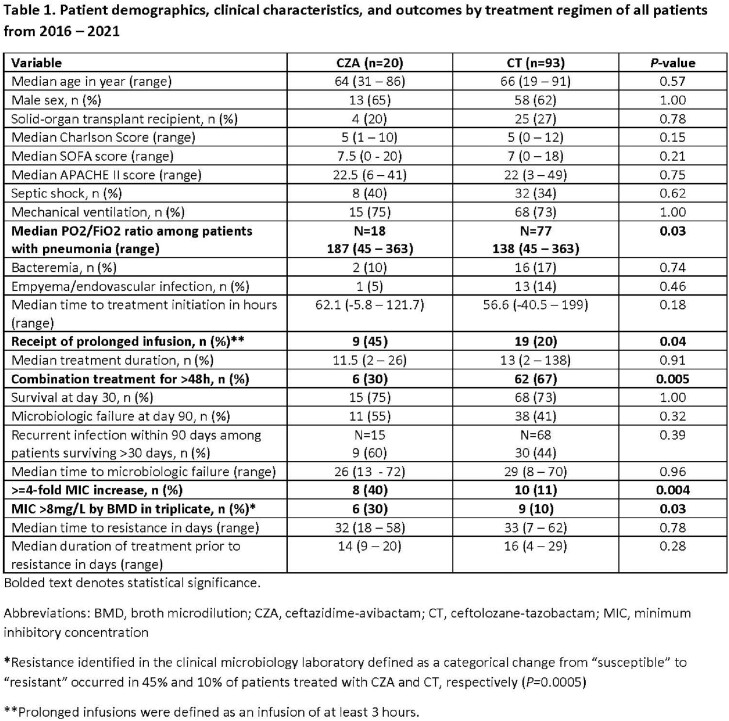

**Figure:**
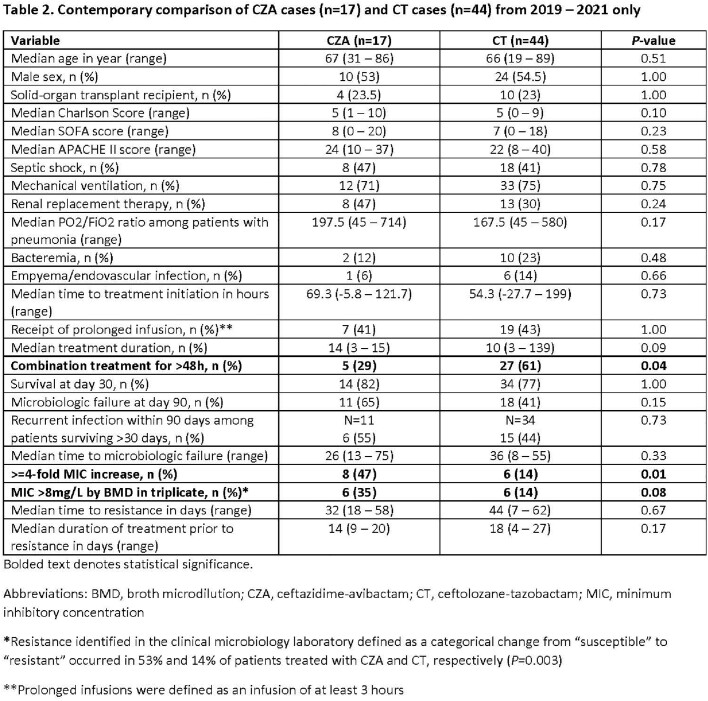

**Figure:**
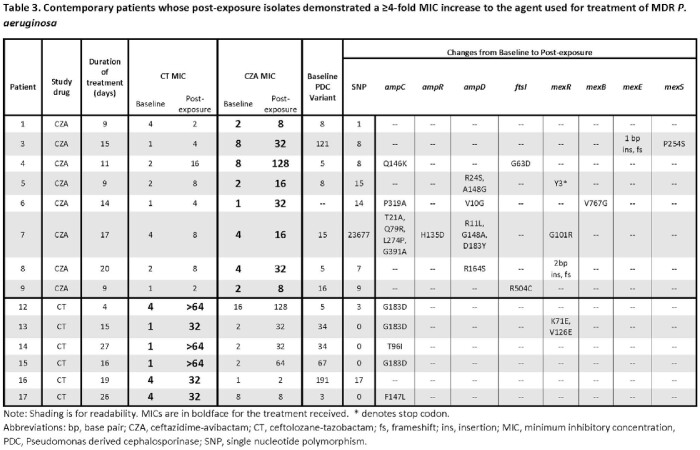

**Conclusion:**

In this non-matched, descriptive analysis, tx-emergent resistance occurred more commonly among pts treated with CZA compared to CT for MDR *P. aeruginosa* infections. Future multicenter studies evaluating comparative clinical outcomes are warranted.

**Disclosures:**

**Ghady Haidar, MD**, Allovir: Grant/Research Support|AstraZeneca: Advisor/Consultant|AstraZeneca: Grant/Research Support|Karius: Advisor/Consultant|Karius: Grant/Research Support|NIH: Grant/Research Support **jason M. Pogue, PharmD**, AbbVie: Advisor/Consultant|Entasis: Advisor/Consultant|Ferring: Advisor/Consultant|GSK: Advisor/Consultant|Merck: Advisor/Consultant|Merck: Grant/Research Support|Qpex: Advisor/Consultant|Shionogi: Advisor/Consultant **Erin K. McCreary, PharmD**, Abbvie: Advisor/Consultant|Ferring: Advisor/Consultant|GSK: Honoraria|La Jolla (Entasis): Advisor/Consultant|LabSimply: Advisor/Consultant|Merck: Advisor/Consultant|Shionogi: Advisor/Consultant|Shionogi: Honoraria **Ryan K. Shields, PharmD, MS**, Allergan: Advisor/Consultant|Cidara: Advisor/Consultant|Entasis: Advisor/Consultant|GSK: Advisor/Consultant|Melinta: Advisor/Consultant|Melinta: Grant/Research Support|Menarini: Advisor/Consultant|Merck: Advisor/Consultant|Merck: Grant/Research Support|Pfizer: Advisor/Consultant|Roche: Grant/Research Support|Shionogi: Advisor/Consultant|Shionogi: Grant/Research Support|Utility: Advisor/Consultant|Venatorx: Advisor/Consultant|Venatorx: Grant/Research Support

